# Supplementary material for: A Facile Synthesis and Molecular Characterization of Certain New Anti-Proliferative Indole-Based Chemical Entities
Source: Int J Mol Sci. 2023 Apr 26;24(9):7862. doi: 10.3390/ijms24097862 (PMC10178769; doi:10.3390/ijms24097862)
Supplement: Supplementary file 1 [file ijms-24-07862-s001.zip › 13CNMR-2-4e.pdf]

C13CPD DMSO D:\\ abari 14

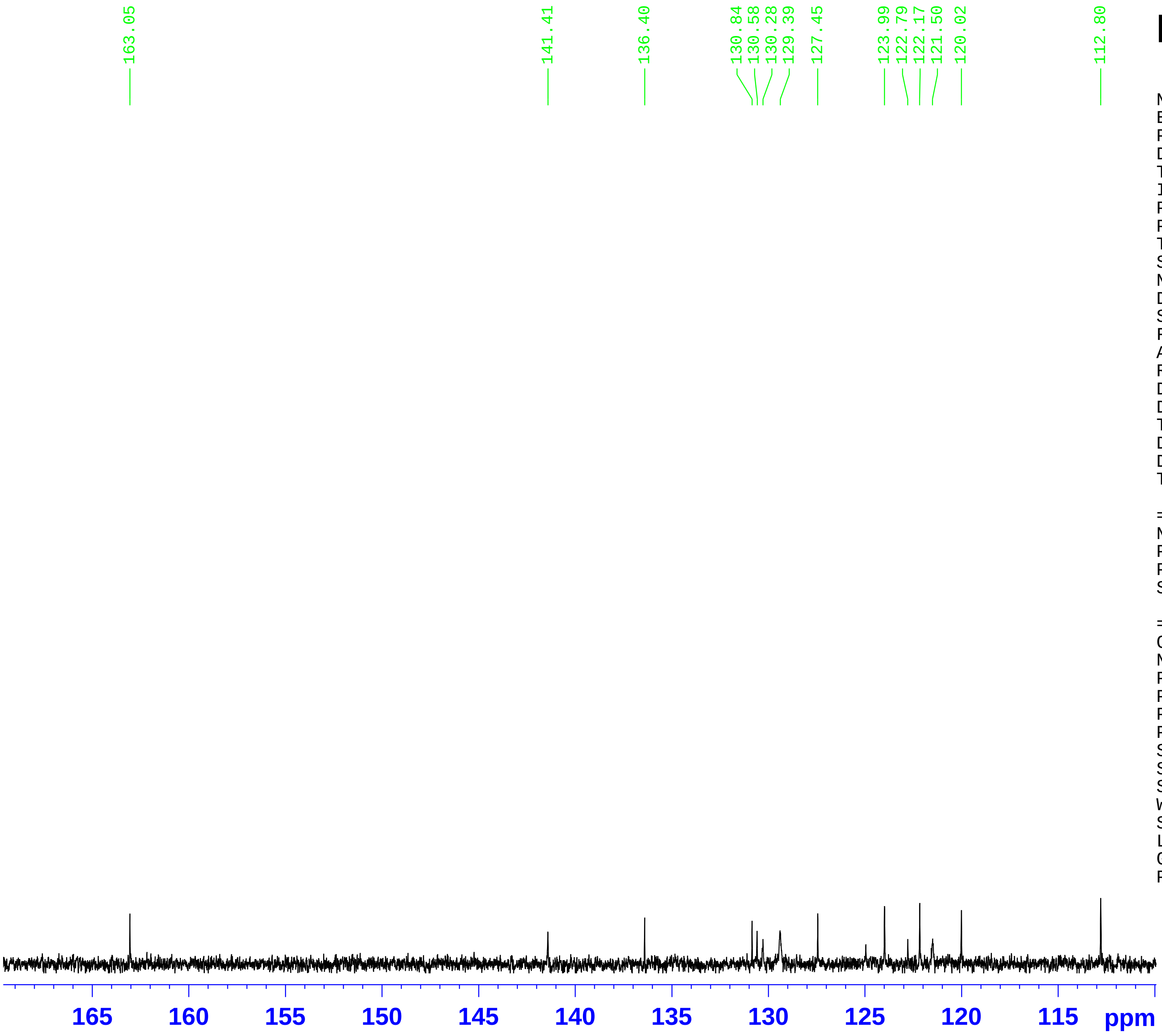

NAME	drattia-RMI-106	
EXPNO	11	
PROCNO	1	
Date_	20160503	
Time	9.30	
INSTRUM	spect	
PROBHD	5 mm PABBO BB-	
PULPROG	zgpg30	
TD	65536	
SOLVENT	DMSO	
NS	4000	
DS	4	
SWH	30030.029	Hz
FIDRES	0.458222	Hz
AQ	1.0912410	sec
RG	2580.3	
DW	16.650	usec
DE	6.50	usec
TE	300.0	K
D1	2.00000000	sec
D11	0.03000000	sec
TD0	1	

======== CHANNEL f1 ========

NUC1	13C	
P1	20.00	usec
PL1	-6.00	dB
SFO1	125.7703643	MHz

======== CHANNEL f2 ========

CPDPRG2	waltz16	
NUC2	1H	
PCPD2	80.00	usec
PL2	-1.10	dB
PL12	13.44	dB
PL13	16.40	dB
SFO2	500.1320005	MHz
SI	32768	
SF	125.7577890	MHz
WDW	EM	
SSB	0	
LB	1.00	Hz
GB	0	
PC	1.40	
