# Supplementary material for: A Facile Synthesis and Molecular Characterization of Certain New Anti-Proliferative Indole-Based Chemical Entities
Source: Int J Mol Sci. 2023 Apr 26;24(9):7862. doi: 10.3390/ijms24097862 (PMC10178769; doi:10.3390/ijms24097862)
Supplement: Supplementary file 1 [file ijms-24-07862-s001.zip › 13CNMR-4e.pdf]

C13CPD DMSO D:\\ abari 14

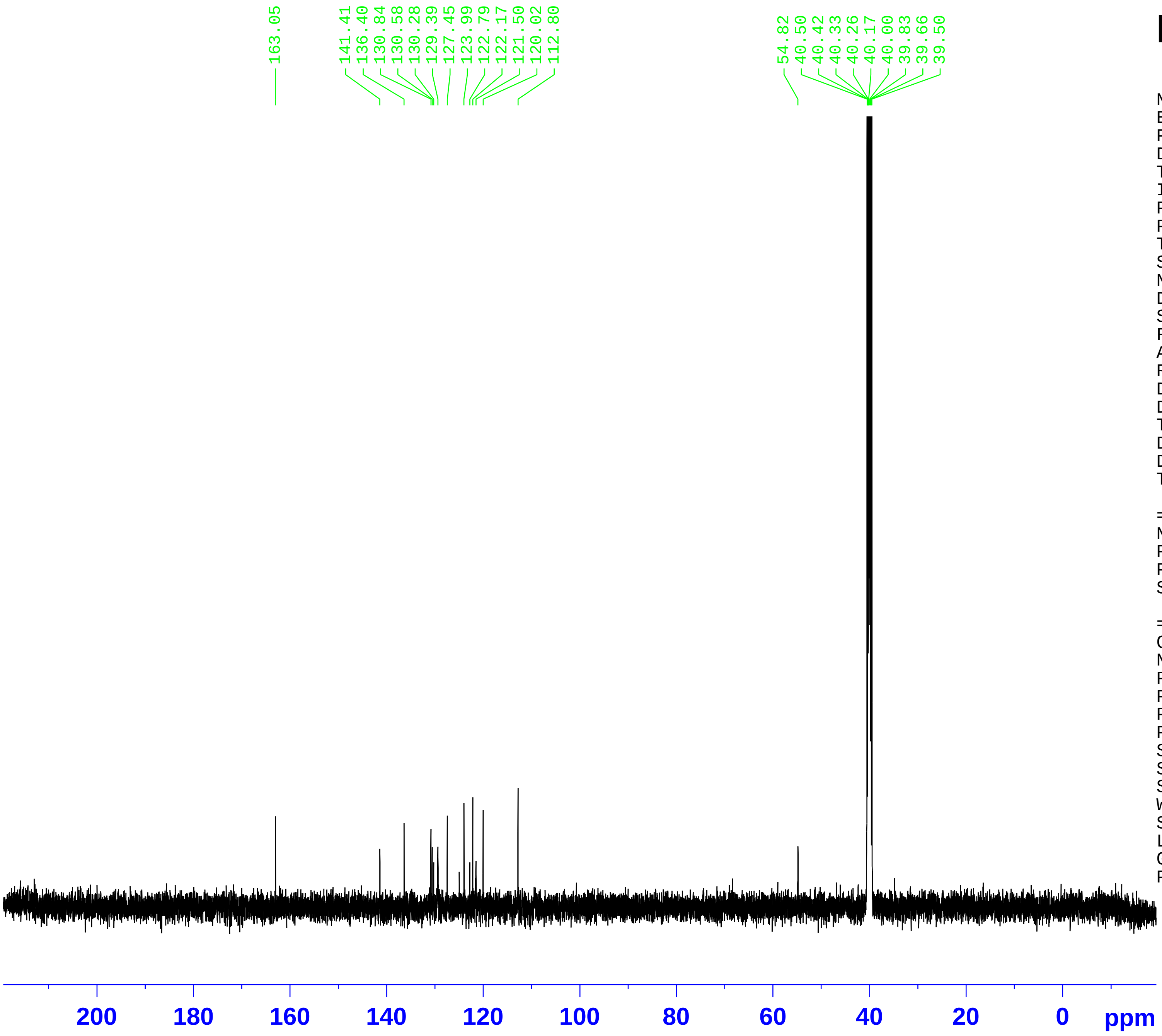

NAME drattia-RMI-106
EXPNO 11
PROCNO 1
Date_ 20160503
Time 9.30
INSTRUM spect
PROBHD 5 mm PABBO BB-
PULPROG zgpg30
TD 65536
SOLVENT DMSO
NS 4000
DS 4
SWH 30030.029 Hz
FIDRES 0.458222 Hz
AQ 1.0912410 sec
RG 2580.3
DW 16.650 usec
DE 6.50 usec
TE 300.0 K
D1 2.00000000 sec
D11 0.03000000 sec
TD0 1

======== CHANNEL f1 ========
NUC1 13C
P1 20.00 usec
PL1 -6.00 dB
SFO1 125.7703643 MHz

======== CHANNEL f2 ========
CPDPRG2 waltz16
NUC2 1H
PCPD2 80.00 usec
PL2 -1.10 dB
PL12 13.44 dB
PL13 16.40 dB
SFO2 500.1320005 MHz
SI 32768
SF 125.7577890 MHz
WDW EM
SSB 0
LB 1.00 Hz
GB 0
PC 1.40
