# Supplementary material for: A Facile Synthesis and Molecular Characterization of Certain New Anti-Proliferative Indole-Based Chemical Entities
Source: Int J Mol Sci. 2023 Apr 26;24(9):7862. doi: 10.3390/ijms24097862 (PMC10178769; doi:10.3390/ijms24097862)
Supplement: Supplementary file 1 [file ijms-24-07862-s001.zip › HNMR-2-4e.pdf]

PROTON DMSO D:\\ abari 14

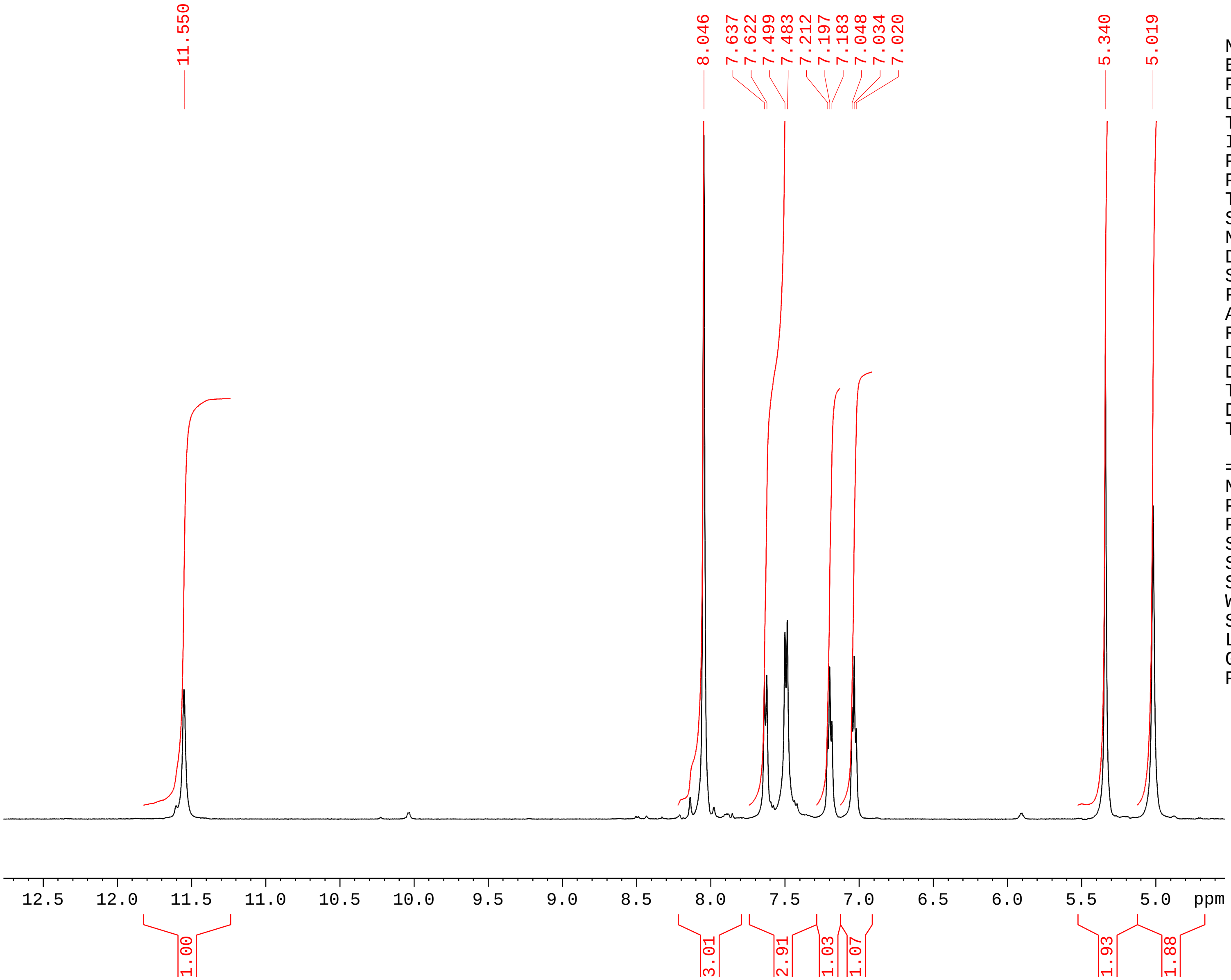

NAME	drattia-RMI-106	
EXPNO	10	
PROCNO	1	
Date_	20160503	
Time	7.30	
INSTRUM	spect	
PROBHD	5 mm PABBO BB-	
PULPROG	zg30	
TD	65536	
SOLVENT	DMSO	
NS	16	
DS	2	
SWH	10330.578	Hz
FIDRES	0.157632	Hz
AQ	3.1720407	sec
RG	181	
DW	48.400	usec
DE	6.50	usec
TE	300.0	K
D1	1.00000000	sec
TD0	1	

======== CHANNEL f1 ========

NUC1	1H	
P1	14.70	usec
PL1	-1.10	dB
SFO1	500.1330885	MHz
SI	32768	
SF	500.1300000	MHz
WDW	EM	
SSB	0	
LB	0.30	Hz
GB	0	
PC	1.00	
