# Supplementary material for: A Facile Synthesis and Molecular Characterization of Certain New Anti-Proliferative Indole-Based Chemical Entities
Source: Int J Mol Sci. 2023 Apr 26;24(9):7862. doi: 10.3390/ijms24097862 (PMC10178769; doi:10.3390/ijms24097862)
Supplement: Supplementary file 1 [file ijms-24-07862-s001.zip › HNMR-2-4f.pdf]

PROTON DMSO D:\\ abari 13

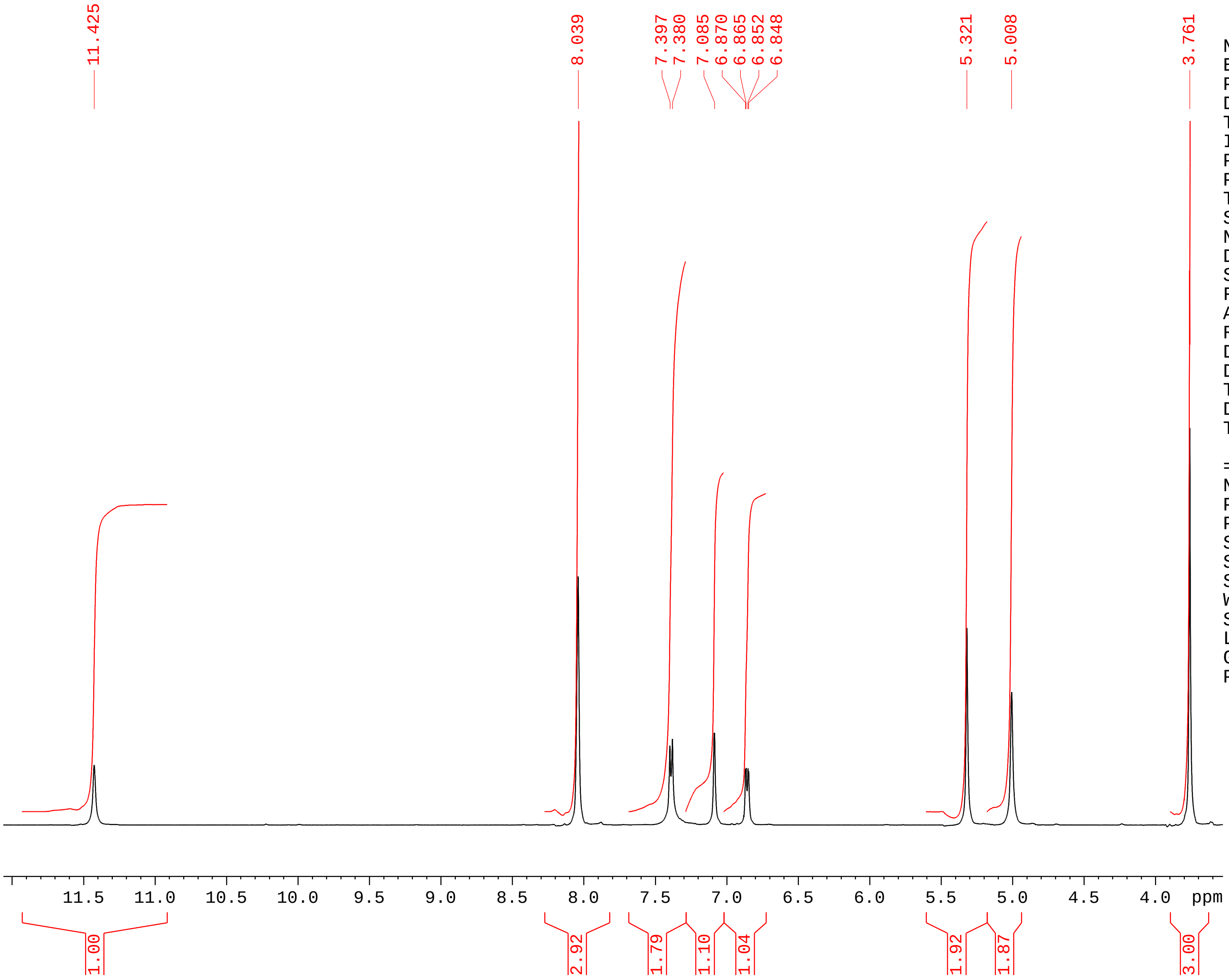

NAME	drattia-RMI-105
EXPNO	10
PROCNO	1
Date_	20160503
Time	3.49
INSTRUM	spect
PROBHD	5 mm PABBO BB-
PULPROG	zg30
TD	65536
SOLVENT	DMSO
NS	16
DS	2
SWH	10330.578 Hz
FIDRES	0.157632 Hz
AQ	3.1720407 sec
RG	181
DW	48.400 usec
DE	6.50 usec
TE	300.0 K
D1	1.00000000 sec
TD0	1

======== CHANNEL f1 ========

NUC1	1H
P1	14.70 usec
PL1	-1.10 dB
SFO1	500.1330885 MHz
SI	32768
SF	500.1300000 MHz
WDW	EM
SSB	0
LB	0.30 Hz
GB	0
PC	1.00
